# Stress and Internalizing Problems in Adolescents: A Dynamic Longitudinal Analysis

**DOI:** 10.3390/jpm15120612

**Published:** 2025-12-08

**Authors:** Filipa Ćavar Mišković, Maja Ribar, Daniela Šupe Domić, Petra Dumanić, Goran Milas

**Affiliations:** 1 Institute of Social Sciences “Ivo Pilar”, 10000 Zagreb, Croatia; filipa.cavar@pilar.hr (F.Ć.M.); maja.ribar@pilar.hr (M.R.); 2 Department of Medical Laboratory Diagnostics, University Hospital of Split, 21000 Split, Croatia; dsupe@kbsplit.hr (D.Š.D.); pdumanic@kbsplit.hr (P.D.); 3 Department of Health Studies, University of Split, 21000 Split, Croatia

**Keywords:** subjective stress, internalizing problems, adolescents, mental health, longitudinal, emotional regulation, dispositional vulnerability

## Abstract

**Background/Objectives:** Internalizing problems commonly increase during adolescence, yet the precise nature of their reciprocal relationship with stress remains unclear. The present study aimed to clarify the directionality of this association by disentangling stable dispositional influences from dynamic, within-person processes. Specifically, we examined whether stress and internalizing symptoms exhibit bidirectional effects over time or are primarily shaped by enduring individual differences. **Methods:** A large, representative sample of 1618 secondary school students (671 males, 947 females; M = 16.3 years) completed measures of subjective stress, emotional problems, and peer problems across three time points spaced six months apart. Data were analyzed using the Random Intercept Cross-Lagged Panel Model (RI-CLPM), which separates stable between-person variance from within-person fluctuations. Model fit was assessed using established criteria (CFI, TLI, RMSEA). **Results:** Subjective stress and emotional problems were strongly associated, whereas the relationship between stress and peer problems was weaker. In both domains, associations were largely explained by stable, trait-like individual differences. All cross-lagged effects at the within-person level were non-significant, indicating no dynamic, time-ordered influence between constructs. These findings provide no empirical support for the stress sensitization or stress generation hypotheses but are consistent with diathesis–stress models emphasizing enduring dispositional vulnerability. **Conclusions:** The results suggest that the link between stress and internalizing symptoms during adolescence primarily reflects stable personality-based factors, such as neuroticism or emotional instability, rather than reciprocal causal processes. Preventive interventions should target emotional regulation and resilience to mitigate the impact of dispositional vulnerabilities on adolescent mental health.

## 1. Introduction

### 1.1. Internalizing Problems in Adolescence

Internalizing problems during childhood and adolescence are characterized by inwardly directed behaviors that include symptoms of anxiety, depression, and social withdrawal [1]. Although such symptoms may resemble clinical affective disorders, they are often expressed at milder, subclinical levels. Research suggests that internalizing and externalizing problems are developmentally intertwined, with each potentially serving as a precursor or risk factor for later psychopathology [2]. While behavioral problems occur across developmental stages, internalizing problems often emerge as the predominant mental health concern during adolescence [3], marking a key risk period for future psychological difficulties [4].

Adolescence is a critical and turbulent phase of development characterized by rapid biological, cognitive, and social changes that expose young people to novel stressors and heightened emotional demands [5,6]. These challenges, combined with evolving interpersonal dynamics and increased autonomy, contribute to an elevated risk of mental health problems [7]. Internalizing symptoms frequently increase during this period [8], and difficulties encountered in adolescence may exert long-lasting negative effects on psychological well-being [9].

Over the past two decades, anxiety and depression rates among adolescents have risen steadily [10,11]. The prevalence of depressive symptoms indicative of risk for clinical depression has increased from approximately 24% in the early 2000s to 37% in recent years [12]. The COVID-19 pandemic further exacerbated this trend, doubling previous prevalence estimates [13]. Recent evidence indicates a concerning upward trend in internalizing symptoms among adolescents in Croatia. For example, a study in Zagreb reported that severe depressive symptoms rose from 15% in 2016 to over 20% in 2021, while anxiety symptoms more than doubled (from 13% to 33%) across the same period [14]. Post-pandemic evidence indicates that elevated anxiety and depressive symptoms have persisted globally; however, patterns across gender are mixed, with some studies reporting comparable rates [15], while recent meta-analytic work [16] shows that increases in depression symptoms have been most consistently observed among female adolescents. Currently, one in five adolescents experience clinically significant anxiety, while approximately one in four exhibit elevated depressive symptoms [13]. These internalizing difficulties typically emerge in middle childhood and peak during late adolescence. Among them, generalized anxiety disorder has become the most common mental health issue in this age group [17].

Stress is widely recognized as a major contributor to the development of internalizing problems [18,19]. However, adolescence represents a period of unique vulnerability due to ongoing neurobiological maturation, identity formation, and environmental transitions. Consequently, the interplay between stress exposure and the emergence of internalizing symptoms remains complex and not fully understood [20,21].

### 1.2. Theoretical Background

#### 1.2.1. The Diathesis–Stress Model

The diathesis–stress model provides a foundational framework for understanding the onset of mental health problems. It posits that psychopathology arises from the interaction between inherent vulnerabilities (biological, cognitive, or personality-based diatheses) and exposure to stressors [22]. Individuals with a predisposition toward emotional instability are more likely to develop psychopathological symptoms when confronted with significant or chronic stress [23,24,25]. Within this model, stress functions as a precipitating factor that activates latent vulnerabilities, contributing to the development of internalizing symptoms [26].

A related perspective—the differential susceptibility hypothesis—suggests that certain individuals are more responsive to environmental influences in general [27]. Rather than being simply vulnerable to negative contexts, such individuals may also benefit more profoundly from positive environments. Nonetheless, under conditions of high or chronic stress, both frameworks predict increased vulnerability to internalizing problems.

#### 1.2.2. Stress Sensitization and Kindling Models

The stress sensitization hypothesis, derived from the kindling model [28], offers a developmental explanation for the progressive worsening of stress-related psychopathology. Initially, significant life stressors may trigger the first onset of internalizing symptoms, but over time, individuals become increasingly sensitive, such that minor stressors can elicit similar reactions [29]. Two main mechanisms are proposed: (1) gradual sensitization through cumulative stress exposure that lowers the threshold for symptom activation and (2) early adversity that predisposes individuals to heightened reactivity to later stressors.

Empirical evidence supports this model, particularly in relation to depression and bipolar disorder [30,31]. However, most studies have examined clinical samples, leaving it unclear whether similar processes operate among adolescents without formal diagnoses or whether stress sensitization contributes to subclinical internalizing symptoms.

#### 1.2.3. Stress Generation and Reciprocal Causation

Contrasting the stress sensitization framework, the stress generation model posits that individuals with existing emotional difficulties actively contribute to the creation of stressful situations [32]. Thus, rather than stress causing symptoms, internalizing problems generate stress—a reversal of causal direction. Despite this distinction, both perspectives may operate simultaneously, forming a reciprocal causation cycle in which stress and internalizing symptoms mutually reinforce each other [19]. This reciprocal dynamic may explain the persistence and escalation of internalizing problems over time.

While both stress sensitization and stress generation models describe links between stress and internalizing problems, they differ in causal emphasis. Stress sensitization models focus on how repeated or early-life stress lowers the threshold for emotional reactivity, such that subsequent stressors—sometimes minor—elicit stronger internalizing responses [29]. In contrast, stress generation models emphasize the role of the individual in actively creating or amplifying stressors through behaviors or interpersonal dynamics associated with existing emotional difficulties [33,34]. Importantly, these models are not mutually exclusive: individuals who are sensitized to stress may also engage in behaviors that generate further stress, creating a reciprocal feedback loop that can maintain or exacerbate internalizing problems over time.

The notion of such bidirectional associations is well supported within developmental psychopathology [32]. Situating these perspectives within adolescent research is particularly important, as adolescence is characterized by rapid social, emotional, and cognitive change that simultaneously increases exposure to stressors and heightens vulnerability to stress reactivity. By estimating within-person cross-lagged associations, the present study seeks to capture both the sensitization effect of stress on later internalizing symptoms and the stress generation effect of internalizing symptoms on subsequent stress.

#### 1.2.4. Insights from Longitudinal Research

Cross-sectional studies consistently report moderate to strong correlations between stress and internalizing symptoms. However, longitudinal findings are more nuanced, generally indicating moderate associations that weaken once temporal ordering and stable individual differences are considered [35]. While some studies find limited support for stress sensitization or generation effects, results remain inconsistent, partly due to methodological variability and small sample sizes. Evidence for reciprocal causality has been observed in a few studies [20,36], yet these effects are often modest and not uniform across different internalizing domains. Moreover, meta-analytic conclusions are constrained by the small number of available longitudinal investigations, highlighting the need for more rigorous, dynamic analyses that can distinguish between stable trait effects and time-specific processes.

### 1.3. The Present Study

Despite growing recognition of internalizing problems as a major public health concern among adolescents, the mechanisms linking stress and internalizing symptoms remain incompletely understood [20]. Competing theoretical models variously conceptualize stress as a cause, consequence, or correlate of internalizing difficulties. To address this ambiguity, the present study utilized a three-wave longitudinal design and employed the Random Intercept Cross-Lagged Panel Model (RI-CLPM), which allows for the separation of stable between-person variance from dynamic within-person changes [37].

The study pursued two primary research aims:

1. To determine the extent to which the association between stress and internalizing problems can be attributed to stable dispositional traits at the between-person level.
2. To examine the strength and direction of within-person effects between stress and internalizing problems across time.

### 1.4. Hypotheses

-
  **H1:**  Because both stress susceptibility and internalizing symptoms demonstrate trait-like stability, a substantial portion of their association will occur at the between-person level, consistent with the diathesis–stress model.
-
  **H2:**  Stress will significantly predict internalizing problems at the within-person level (stress sensitization), with a smaller but notable reciprocal effect from internalizing problems to stress (stress generation), reflecting stress sensitization, stress generation, and reciprocal causation processes.

## 2. Method

### 2.1. Participants and Procedure

A three-wave longitudinal design was employed to explore the dynamic relationship between subjective stress and internalizing problems. Data were collected across three waves between spring 2022 and spring 2023 from students enrolled in 17 public secondary schools in Zagreb, Croatia. The study formed part of the Longitudinal Adolescent Stress Study.

Croatia’s secondary education system comprises gymnasiums (general academic), vocational, and art schools. Because of their small population and potential dual enrollment, art schools were excluded. Schools were selected using proportional random sampling based on enrollment data, stratified by curricular orientation. Initially, 18 vocational and 10 gymnasiums were identified; 15 schools participated in Wave 1, with two joining in Wave 2, for a total of 17 schools (10 vocational and 7 gymnasiums). Within each school, five first-year and five second-year classes were included.

To justify the adequacy of the sample for the planned RI-CLPM analyses, we conducted a Monte-Carlo power simulation using the powRICLPM package (version 0.2.1) [38]. We simulated 24 experimental conditions that varied by sample size, while holding constant key model parameters informed by prior work, including: (a) intraclass correlations (ICC = 0.40), (b) random-intercept covariance (r = 0.60), (c) within-person residual correlation (r = 0.25), (d) measurement reliability (λ = 0.90), and (e) three measurement waves. Each condition was based on 1000 replications at α = 0.05. Results indicated that a sample size of approximately 1500 participants would be required to achieve conventional power (0.80) to detect moderate within-person cross-lagged effects. Our final analytic sample (*N* = 1618) exceeded this estimated requirement, suggesting that the study was adequately powered for the primary analyses.

The final analytic sample included 1618 students who provided valid data in at least two of the three waves. Vocational students comprised approximately 60% of the sample, consistent with national distributions [39]. Gender representation was approximately balanced, with a slight overrepresentation of female and gymnasium students. Data were collected approximately six months apart (March 2022, October 2022, and April 2023). The socio-demographic composition of the sample is presented in Table 1.

Prior to participation, all students received information about study aims and procedures. Written informed consent was obtained from participants, and parental or guardian consent was secured for students younger than 15 years.

### 2.2. Measures

#### 2.2.1. Subjective Stress

Subjective stress was assessed using a Croatian adaptation [40] of the Problem Questionnaire [41]. The measure includes 18 items covering six domains of adolescent life: school, future, family, peers, romantic relationships, and self. Participants rated the stressfulness of each situation on a 5-point Likert scale ranging from 1 (“Not stressful at all”) to 5 (“Extremely stressful”). Internal consistency was excellent across all waves (α = 0.91–0.92).

#### 2.2.2. Internalizing Problems

Internalizing problems were measured using the Croatian version of the Strengths and Difficulties Questionnaire (SDQ) [42]. Two subscales—Emotional Symptoms and Peer Problems—were used to form the composite Internalizing Problems index. Internal consistency was acceptable for the composite score (α = 0.71–0.75) and good for Emotional Symptoms (α = 0.73–0.77). The Peer Problems subscale showed lower reliability (α = 0.51–0.53), consistent with prior research [43].

#### 2.2.3. Analytic Strategy

Missing data ranged from 18.6% to 26.8% across waves, primarily due to student absences or incomplete responses. Little’s MCAR test indicated data were not missing completely at random (χ^2^[74] = 110.67, *p* = 0.004). As the missing-at-random (MAR) assumption cannot be directly tested—since it pertains to unobserved data—we used indirect methods to evaluate the plausibility of the data being not missing at random (MNAR). Specifically, we employed the pattern mean difference approach [44] and conducted binomial regression analyses to investigate whether missingness in any wave was associated with subjective stress or internalizing problem scores from other waves. After applying the Bonferroni correction (adjusted threshold *p* = 0.0028), none of the 18 analyses yielded a statistically significant odds ratio (Table S1 in Supplementary Materials). We further assessed the plausibility of the MAR assumption by regressing the missingness indicators at Waves 2 and 3 on sex, SES, and school track. The proportion of explained variance ranged from 0.01 to 0.12, indicating that missingness was partially predictable from observed variables (Table S2 in the Supplementary Materials). We attempted to include these variables as auxiliary variables in the RI-CLPM; however, the model failed to converge due to a Heywood case. Consequently, the RI-CLPM analyses were conducted without auxiliary variables, and the MAR assumption should therefore be interpreted with this limitation in mind. Missingness was therefore handled using Full Information Maximum Likelihood (FIML) estimation.

To evaluate measurement invariance, we conducted longitudinal (configural, metric, and scalar) and sex invariance tests for the SDQ subscales and the subjective stress measure, following established guidelines [45]. Model fit was evaluated using ΔCFI and ΔRMSEA criteria, with a change in CFI ≤ 0.01 and a change in RMSEA ≤ 0.015 indicating acceptable invariance.

To examine reciprocal associations between stress and internalizing problems, we applied a Random Intercept Cross-Lagged Panel Model (RI-CLPM) [37]. Although the RI-CLPM is useful for disentangling stable between-person differences from within-person fluctuations over time [46], its application requires several assumptions—such as within-person stationarity and adequate measurement invariance—that warrant careful consideration. In the present study, the use of the RI-CLPM is consistent with the dispositional stability of both internalizing symptoms and stress susceptibility; however, the model’s ability to capture dynamic processes is constrained by the three-wave design and the relatively short developmental window. These limitations mean that parameter estimates, including autoregressive and cross-lagged effects, may be less precise and potentially sensitive to temporal heterogeneity. To maintain model parsimony, we imposed equality constraints on autoregressive and cross-lagged paths, but we acknowledge that with only three waves such constraints may obscure meaningful variation across time and should ideally be evaluated after establishing strict measurement invariance. We have therefore interpreted the RI-CLPM results cautiously and in light of these methodological constraints.

Because the present study covered a relatively short developmental window during adolescence—and consistent with theoretical expectations of temporal stability in these processes—we constrained the autoregressive and cross-lagged paths to equality across waves. This decision enhanced model parsimony and yielded more stable parameter estimates. To evaluate the appropriateness of these equality constraints, two nested RI-CLPMs were compared: one with all paths freely estimated and another with the corresponding paths constrained to be equal across time. Model comparison relied on both statistical and practical fit criteria. Statistically, chi-square difference tests (Δχ^2^) were used to assess potential reductions in model fit, with a *p* value below 0.05 indicating significant deterioration [47]. Given the test’s sensitivity to large samples, we also examined changes in alternative fit indices following the recommendations of Cheung and Rensvold [45]. A decrease of ≥0.01 in the Comparative Fit Index (CFI) or Tucker–Lewis Index (TLI), or an increase of ≥0.015 in the Root Mean Square Error of Approximation (RMSEA), was interpreted as evidence of meaningful decline in model fit. The more parsimonious constrained model was retained only when these thresholds indicated no substantial loss of fit.

The overall fit of the retained model was evaluated using multiple indices: the chi-square (χ^2^) statistic to assess the discrepancy between the observed and model-implied covariance matrices, along with the TLI, CFI, and RMSEA. Following established conventions, model fit was considered acceptable when TLI and CFI values approached or exceeded 0.95 and RMSEA values were below 0.07 [48,49]. All statistical analyses were conducted using SPSS 27 [50] and AMOS 27 [51].

## 3. Results

### Descriptive Statistics

The observed mean scores (see Table 2) for internalizing problems and their components were generally consistent with normative data for adolescents of comparable age [52]. Similarly, mean subjective stress levels aligned with previously reported adolescent norms [40]. No systematic trends or marked fluctuations were observed across the three measurement waves. The stress scale demonstrated excellent internal consistency, whereas the composite internalizing problems and emotional problems scales showed satisfactory reliability. The peer problems scale exhibited lower reliability—comparable to that reported in the original validation study by Goodman et al. [52]—but remained acceptable for inclusion in subsequent analyses.

As expected, correlations between repeated measures of the same constructs across waves were high (see Table 3). Test–retest reliabilities ranged from 0.71 to 0.81 for subjective stress, 0.70 to 0.78 for emotional problems, and 0.57 to 0.71 for peer problems, indicating moderate to strong temporal stability. Associations between subjective stress and emotional problems were also robust, with correlations ranging from 0.56 to 0.70 across waves. In contrast, correlations between subjective stress and peer problems were weaker (0.24 to 0.38), likely reflecting both conceptual differences and the reduced reliability of the peer problems subscale.

Prior to conducting the main longitudinal analyses, both longitudinal and sex invariance were established for all included instruments. Configural, metric, and scalar invariance were confirmed, ensuring that observed changes over time reflect true variation rather than shifts in measurement properties. The results of these tests are reported in Tables S3 and S4 in the Supplementary Materials.

To further examine temporal dynamics, two sets of nested Random Intercept Cross-Lagged Panel Models (RI-CLPMs) were compared: one with all autoregressive and cross-lagged paths freely estimated, and another with corresponding paths constrained to equality across waves. In both cases, model comparisons indicated no meaningful differences in overall fit (see Supplementary Tables S5 and S6). Although a statistically significant chi-square difference emerged, this effect likely reflected the large sample size rather than substantive model misfit. Quantitatively, the constrained models showed minimal changes in fit indices (ΔCFI ≤ 0.002; ΔTLI ≤ 0.001; ΔRMSEA ≤ 0.003) and retained acceptable absolute fit across all indices. Because the principal fit indices (CFI, TLI, and RMSEA) showed no appreciable deterioration, the more parsimonious constrained models were retained. This decision was further supported by theoretical expectations of temporal stability and the enhanced precision of parameter estimates under constrained specifications. Full model specifications are provided in Supplementary Tables S7 and S9.

**Figure 1:**
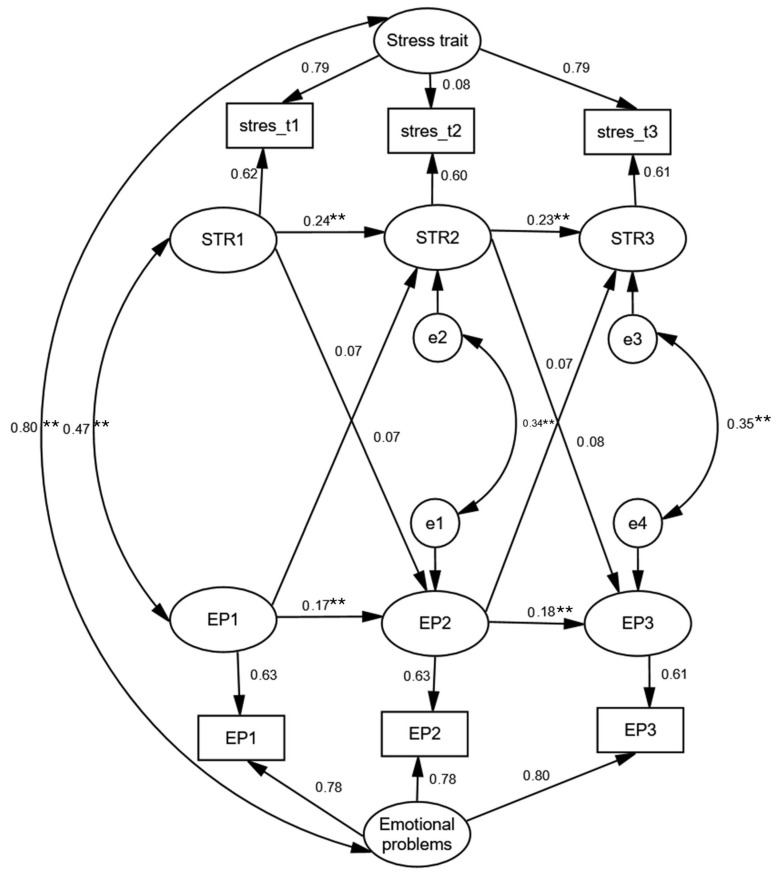
Standardized Random Intercept Cross-Lagged Panel Model Solution Describing the Longitudinal Relationship Between Subjective Stress and Emotional Problems in Adolescents (*N* = 1618). Note: Model fit: χ^2^(1) = 1.662, *p* = 0.197; RMSEA = 0.020 (90% CI 0.000–0.073); CFI = 1.000; TLI = 0.997. ** *p* < 0.01.

As illustrated in Figure 1, the association between subjective stress and emotional problems was primarily driven by stable, trait-like factors that remained highly correlated across time, underscoring the role of enduring dispositional influences in the stress–psychopathology link. Standardized effect sizes (β) are depicted in Figure 1. Complete parameter estimates, including effect sizes and R^2^ values for all endogenous variables, are reported in Supplementary Table S8.

Following Kenny and Zautra’s [53] framework, the autoregressive coefficients, which reflect slowly changing factors or trait-like stability, indicated low temporal stability for both subjective stress and emotional problems, with values ranging from 0.17 to 0.24. This means that when a young person was higher (or lower) than their own usual level at one wave, this deviation tended to fade rather than carry over to the next wave. In other words, short-term within-person fluctuations in these constructs exhibited limited stability over time. Importantly, this finding does not contradict the high test–retest correlations reported earlier. Test–retest correlations capture between-person stability—that is, the degree to which young people maintain their rank ordering relative to one another across waves. By contrast, the low autoregressive coefficients speak to within-person stability of momentary deviations around each individual’s typical level. Thus, it is possible to observe high between-person stability alongside low stability in short-term within-person fluctuations. Nevertheless, wave-specific deviations from the stable trait components of both constructs were moderately correlated across the three waves, with correlations ranging from 0.34 to 0.47, indicating a degree of consistent co-fluctuation over time.

The cross-lagged coefficients derived from the RI-CLPM—which estimate the directional influence of deviations in one construct on subsequent deviations in the other—were all non-significant. These findings indicate that the observed association between subjective stress and emotional problems was predominantly explained by shared variance at the stable, between-person level, rather than by dynamic within-person processes. Consequently, the results provide no empirical support for either the stress generation or stress sensitization hypotheses in this context.

**Figure 2:**
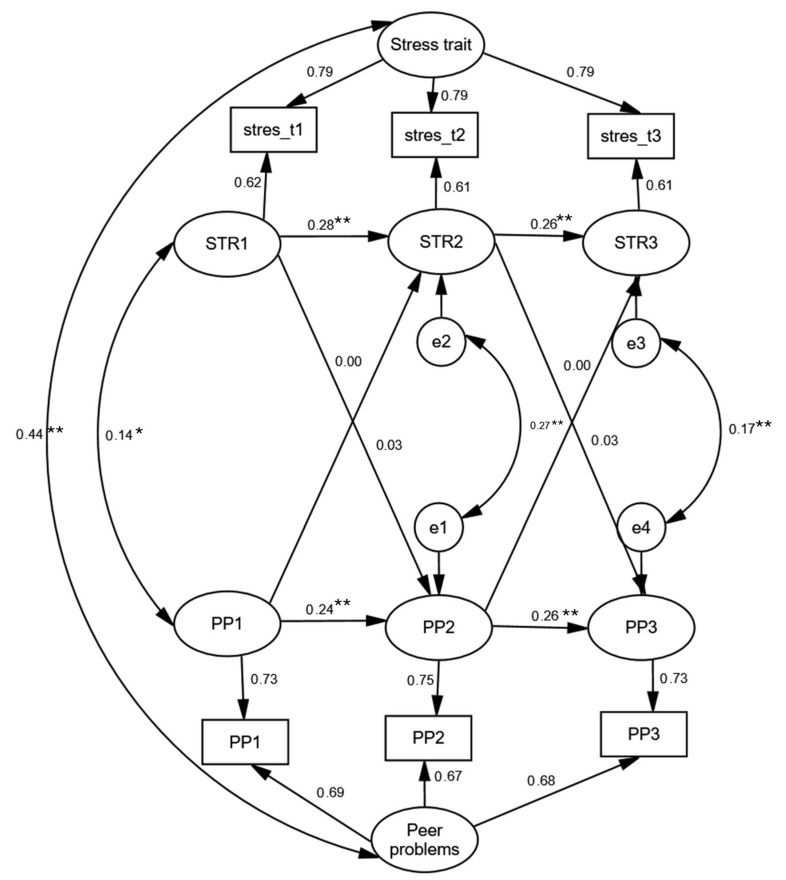
Standardized Random Intercept Cross-Lagged Panel Model Solution Describing the Longitudinal Relationship Between Subjective Stress and Peer Problems in Adolescents (*N* = 1618). Note. Model fit: χ^2^(1) = 3.132, *p* = 0.077; RMSEA = 0.036 (90% CI 0.000–0.085); CFI = 0.999; TLI = 0.984. ** *p* < 0.01; * *p* < 0.05.

As depicted in Figure 2, the longitudinal relationship between subjective stress and peer problems revealed a similar pattern; corresponding full model parameter estimates are reported in Supplementary Table S10. Although the constructs were significantly correlated at the level of stable traits, the association was weaker than that observed between stress and emotional problems (r = 0.44, *p* < 0.001). Autoregressive coefficients reflected modest stability in deviations from the stable trait for both constructs (βs ranging from 0.24 to 0.28), accompanied by small but statistically significant concurrent correlations (rs = 0.14–0.27). Consistent with the findings for emotional problems, none of the cross-lagged paths reached statistical significance, suggesting that fluctuations in stress did not predict subsequent changes in peer problems—or vice versa. Thus, there was no evidence supporting the stress sensitization, stress generation, or reciprocal causation models when peer problems were considered as a component of internalizing difficulties.

## 4. Discussion

Although most theoretical frameworks attribute the onset of internalizing problems during adolescence to exposure to stress [18,19], the precise mechanisms linking these processes remain incompletely understood. To address this gap, the present longitudinal study applied the Random Intercept Cross-Lagged Panel Model (RI-CLPM) to a large sample of high school students. This analytic approach allowed for the separation of stable, between-person differences from dynamic, within-person fluctuations, thereby providing a more rigorous test of the directional associations between stress and internalizing symptoms. Importantly, adolescence represents a sensitive developmental period characterized by heightened neurobiological and social-affective reactivity, driven by maturational changes in limbic and prefrontal systems, which may amplify emotional responses to perceived stress [54].

### 4.1. Summary of Findings

The findings revealed a strong association between subjective stress and emotional problems, consistent with prior work highlighting their conceptual and affective overlap [55]. In contrast, the relationship between stress and peer problems was notably weaker, likely reflecting both the lower reliability of the peer problems subscale and the weaker conceptual correspondence between these constructs.

RI-CLPM analyses demonstrated that the association between stress and emotional problems was predominantly driven by stable, trait-like individual differences. This pattern aligns with dispositional models, which emphasize the role of enduring personality traits—particularly neuroticism—in shaping both stress reactivity and emotional vulnerability [56,57]. The absence of significant cross-lagged effects suggests a lack of dynamic, time-ordered influence between the two constructs, offering no empirical support for the stress generation or stress sensitization hypotheses. Instead, these findings are more consistent with the diathesis–stress framework, which posits that internalizing difficulties emerge from interactions between latent vulnerabilities and external stressors [26].

Similarly, the association between stress and peer problems appeared to reflect primarily dispositional influences, although the proportion of explained variance was smaller. This suggests that the relationship may be driven by a stable underlying factor—potentially neuroticism—which has been linked to interpersonal difficulties [58]. Again, the absence of significant within-person cross-lagged effects provides no support for the stress sensitization, stress generation, or reciprocal causality models when peer problems are considered as a component of internalizing difficulties.

### 4.2. Comparison with Previous Research

These findings diverge in part from prior longitudinal studies reporting bidirectional associations. For instance, Lee [59] found weak but significant reciprocal effects between stress and internalizing symptoms in a Korean adolescent sample, consistent with both sensitization and generation processes. Similarly, Goldstein et al. [60] observed significant cross-lagged associations in a two-wave design. However, both studies relied on traditional Cross-Lagged Panel Models, which do not distinguish between- and within-person variance and thus may overestimate directional effects [46]. By contrast, Jenness et al. [20], using an intensive longitudinal design and multilevel modeling, also detected bidirectional associations, although methodological differences—particularly in measurement timing and analytic strategy—limit direct comparability.

A meta-analysis by March-Llanes et al. [35] similarly reported bidirectional effects but drew upon a relatively small pool of longitudinal studies, many of which employed methods unable to disentangle trait-level and within-person dynamics. Taken together, these inconsistencies underscore the importance of analytic approaches, such as the RI-CLPM, that partition variance sources to clarify whether associations reflect stable dispositions or true temporal processes.

Despite variations across studies, a consistent theme emerges: the relationship between stress and internalizing symptoms appears largely shaped by enduring dispositional traits, such as neuroticism or emotional instability. While this conclusion may seem at odds with evidence supporting stress sensitization or generation models, prior meta-analytic work indicates that such effects, when present, are typically small in magnitude [35,36] and may differ across internalizing subdomains [20]. In interpreting these findings, it is essential to consider the Croatian sociocultural context. Recent evidence from a large Positive Youth Development study demonstrates that Croatian adolescents’ depression, anxiety, and stress are strongly shaped by school and family relational climates, suggesting that stable contextual factors may contribute to the trait-like patterns observed in our data [61].

### 4.3. Methodological Considerations and Future Directions

The absence of cross-lagged effects in the present study may partly reflect the six-month interval between assessments. It is plausible that bidirectional influences, as observed in high-frequency longitudinal studies, are short-lived and dissipate over longer time spans. Future research should therefore employ hybrid designs that combine traditional longitudinal frameworks with intensive data collection techniques, such as ecological momentary assessment (EMA), to capture the dynamic interplay between stress and internalizing symptoms as they unfold in real time.

In addition, incorporating multi-informant perspectives (e.g., parents, teachers, peers) and objective indicators of stress (e.g., event logs, cortisol measures) would enhance ecological validity and reduce the impact of shared method variance. Such methodological triangulation would advance a more comprehensive and fine-grained understanding of adolescent emotional functioning.

### 4.4. Implications for Practice

The findings suggest that subjective stress and internalizing problems co-occur primarily due to stable, dispositional factors, rather than direct causal interactions over time. This underscores the role of emotional stability—the capacity to regulate and maintain affective equilibrium under stress—as a key protective factor during adolescence. Given adolescents’ heightened vulnerability to emotional dysregulation, preventive interventions should prioritize the development of emotional regulation skills. Clinically, the dominance of trait-like associations suggests that adolescents who report persistently elevated subjective stress may represent a high-risk subtype for chronic internalizing psychopathology, consistent with longitudinal evidence showing that early stable distress predicts recurrent emotional disorder into adulthood [62]. From a preventative standpoint, this implies that identifying and supporting youth with stable stress elevations—rather than focusing solely on acute stress fluctuations—may be particularly critical for reducing later psychiatric morbidity.

Evidence supports mindfulness-based interventions as effective strategies for enhancing emotional regulation, reducing automatic reactivity [63], and fostering cognitive reappraisal, a mechanism through which individuals reinterpret stressors in less distressing terms [64,65]. Promoting such competencies may help mitigate the onset and severity of internalizing symptoms and strengthen resilience during this critical developmental period.

### 4.5. Study Limitations

Although this study benefited from a large sample, several limitations should be acknowledged. First, while the longitudinal design facilitated the examination of temporal associations, it does not permit strong causal inference. The six-month spacing between assessments may have been too long to detect short-term within-person fluctuations. Second, potential moderators and mediators, such as emotional stability, resilience, and coping mechanisms, were not explicitly modeled and may have influenced the observed patterns. Third, the exclusive reliance on self-report measures introduces possible biases, including social desirability effects, recall errors, and common method variance [66,67]. Fourth, the study sample consisted of adolescents attending public secondary schools in Zagreb and was close to reflecting the school distribution at baseline; however, attrition led to underrepresentation of vocational-track students in later waves, which may introduce longitudinal selection bias. Fifth, the internal consistency of the SDQ peer problems subscale was modest (α = 0.51–0.53), which may have attenuated observed associations with stress and limits the precision of parameter estimates; findings involving this subscale should therefore be interpreted cautiously. Sixth, because the study relies on a three-wave design with six-month intervals, the precision of the estimated within-person effects is limited, and the analyses may not capture processes that operate on shorter or more variable time scales. In addition, limited waves and sparse subgroup sizes reduced statistical power for multigroup analyses and contributed to convergence problems in more complex models (e.g., including sex, school track, and SES). Finally, although we interpret the findings as reflecting stable, trait-like influences, we acknowledge that our conclusions are tentative. We attempted to estimate an RI-CLPM including emotional stability as a between-person covariate, but this was not technically feasible in AMOS, and the corresponding model in lavaan did not converge; thus, the present results should be regarded as provisional and hypothesis-generating rather than definitive. Future studies should incorporate behavioral observations, physiological stress markers, and contextual measures (e.g., exposure to life events or indices of positive functioning) to broaden interpretive scope, and should include additional waves and larger samples to permit estimation of more complex and potentially heterogeneous models.

## 5. Conclusions

Overall, the findings indicate that the association between subjective stress and internalizing symptoms is largely explained by stable, dispositional factors, providing tentative support for a diathesis–stress interpretation rather than for dynamic, reciprocal causation. These insights highlight the potential value of early, targeted interventions that strengthen emotional regulation and resilience during adolescence. By addressing underlying emotional vulnerabilities, preventive efforts may reduce both the onset and chronicity of internalizing difficulties, contributing to broader efforts to promote adolescent mental health and well-being.

## Figures and Tables

**Table 1:** Socio-demographic Composition of the Sample Across Waves.

		Age		Biological Sex		School Type	
	*N*	M	SD	% Males	% Females	% Vocational	% Gymnasium
Time 1	1308	16.32	0.63	43.27	56.73	62.19	37.81
Time 2	1186	16.83	0.62	40.98	59.02	62.70	37.30
Time 3	1346	17.35	0.62	40.19	59.81	53.71	46.29

**Table 2:** Means and Standard Deviations for Subjective Stress and Internalizing Problems at Each Measurement Point.

	M	SD	Min.	Max.	Skewness	Kurtosis	Reliability (Alpha)
Stress t1	2.21	0.78	1.00	5.00	0.627	−0.314	0.91
Stress t2	2.34	0.77	1.00	4.83	0.356	−0.483	0.91
Stress t3	2.12	0.77	1.00	5.00	0.715	0.103	0.92
Internalizing problems t1	6.02	3.49	0.00	20.00	0.547	−0.245	0.73
Internalizing problems t2	6.36	3.56	0.00	20.00	0.603	−0.025	0.75
Internalizing problems t3	6.32	3.42	0.00	19.00	0.507	−0.158	0.71
Emotional problems t1	3.80	2.54	0.00	10.00	0.477	−0.683	0.77
Emotional problems t2	3.97	2.49	0.00	10.00	0.359	−0.728	0.75
Emotional problems t3	3.81	2.40	0.00	10.00	0.434	−0.595	0.73
Peer problems t1	2.22	1.65	0.00	10.00	0.912	0.868	0.51
Peer problems t2	2.38	1.68	0.00	10.00	0.819	0.595	0.53
Peer problems t3	2.51	1.70	0.00	10.00	0.684	0.187	0.51

**Table 3:** Zero-Order Correlations between Subjective Stress and Emotional and Peer Problems at Three Time Points.

		1	2	3	4	5	6	7	8
1	Stress t1	—							
2	Stress t2	0.749 **	—						
3	Stress t3	0.638 **	0.719 **	—					
4	Emotional problems t1	0.678 **	0.587 **	0.512 **	—				
5	Emotional problems t2	0.537 **	0.642 **	0.525 **	0.700 **	—			
6	Emotional problems t3	0.505 **	0.550 **	0.635 **	0.642 **	0.675 **	—		
7	Peer problems t1	0.298 **	0.215 **	0.274 **	0.336 **	0.237 **	0.249 **	—	
8	Peer problems t2	0.273 **	0.368 **	0.318 **	0.297 **	0.425 **	0.329 **	0.543 **	—
9	Peer problems t3	0.188 **	0.265 **	0.312 **	0.176 **	0.276 **	0.366 **	0.483 **	0.608 **

Note: ** *p* < 0.001.

## Data Availability

The data that support the findings of this study are available from the corresponding author, G.M., upon reasonable request.

## Supplementary Materials

The following supporting information can be downloaded at: https://www.mdpi.com/article/10.3390/jpm15120612/s1. Table S1: Model Fit Comparison Between Unconstrained and Constrained (Autoregressive and Cross-Lagged Paths) RI-CLPM Models: Subjective Stress and Emotional Problems; Table S2: Model Fit Comparison Between Unconstrained and Constrained (Autoregressive and Cross-Lagged Paths) RI-CLPM Models: Subjective Stress and Peer Problems; Table S3: Measurement Invariance Across Sex for Subjective Stress, Emotional Problems, and Peer Problems; Table S4: Longitudinal Measurement Invariance for Subjective Stress, Emotional Problems, and Peer Problems; Table S5: Model Fit Comparison Between Unconstrained and Constrained (Autoregressive and Cross-Lagged Paths) RI-CLPM Models: Subjective Stress and Emotional Problems; Table S6: Model Fit Comparison Between Unconstrained and Constrained (Autoregressive and Cross-Lagged Paths) RI-CLPM Models: Subjective Stress and Peer Problems; Table S7: Random Intercept Cross-Lagged Panel Model (RI-CLPM) Specification for Stress and Emotional Problems; Table S8: Parameter Estimates for the RI-CLPM: Stress and Emotional Problems; Table S9: Random Intercept Cross-Lagged Panel Model (RI-CLPM) Specification for Stress and Peer Problems; Table S10: Parameter Estimates for the RI-CLPM: Stress and Peer Problems.

## References

[1] D’Urso G., Symonds J. (2022). Developmental Cascades of Internalizing and Externalizing Problems from Infancy to Middle Childhood: Longitudinal Associations with Bullying and Victimization. J. Sch. Violence.

[2] Oh Y., Greenberg M.T., Willoughby M.T., Vernon-Feagans L., Blair C.B., Burchinal M.R., Cox M., Garrett-Peters P.T., Frank J.L., Mills-Koonce W.R. (2020). Examining Longitudinal Associations between Externalizing and Internalizing Behavior Problems at Within- and Between-Child Levels. J. Abnorm. Child Psychol..

[3] Black L., Panayiotou M., Humphrey N. (2022). Internalizing Symptoms, Well-Being, and Correlates in Adolescence: A Multiverse Exploration via Cross-Lagged Panel Network Models. Dev. Psychopathol..

[4] Oerlemans A.M., Wardenaar K.J., Raven D., Hartman C.A., Ormel J. (2020). The Association of Developmental Trajectories of Adolescent Mental Health with Early-Adult Functioning. PLoS ONE.

[5] Arnett J. (1999). Adolescent Storm and Stress, Reconsidered. Am. Psychol..

[6] Arnett J.J. (2000). Emerging Adulthood: A Theory of Development from the Late Teens through the Twenties. Am. Psychol..

[7] Blakemore S.J. (2019). Adolescence and Mental Health. Lancet.

[8] Thapar A., Collishaw S., Pine D.S., Thapar A.K. (2012). Depression in Adolescence. Lancet.

[9] Clayborne Z.M., Varin M., Colman I. (2019). Systematic Review and Meta-Analysis: Adolescent Depression and Long-Term Psychosocial Outcomes. J. Am. Acad. Child Adolesc. Psychiatry.

[10] Twenge J.M. (2020). Increases in Depression, Self-Harm, and Suicide Among U.S. Adolescents After 2012 and Links to Technology Use: Possible Mechanisms. Psychiatr. Res. Clin. Pract..

[11] Keyes K.M., Platt J.M. (2024). Annual Research Review: Sex, Gender, and Internalizing Conditions among Adolescents in the 21st Century—Trends, Causes, Consequences. J. Child Psychol. Psychiatry.

[12] Shorey S., Ng E.D., Wong C.H.J. (2022). Global Prevalence of Depression and Elevated Depressive Symptoms among Adolescents: A Systematic Review and Meta-Analysis. Br. J. Clin. Psychol..

[13] Racine N., McArthur B.A., Cooke J.E., Eirich R., Zhu J., Madigan S. (2021). Global Prevalence of Depressive and Anxiety Symptoms in Children and Adolescents during COVID-19: A Meta-Analysis. JAMA Pediatr..

[14] Ajduković M., Kožljan P. (2023). Depression, Anxiety and Stress of Adolescents Before and During the Fourth Wave of the COVID-19 Pandemic. Soc. Psihijatr..

[15] Samji H., Wu J., Ladak A., Vossen C., Stewart E., Dove N., Long D., Snell G. (2022). Review: Mental Health Impacts of the COVID-19 Pandemic on Children and Youth—A Systematic Review. Child Adolesc. Ment. Health.

[16] Madigan S., Racine N., Vaillancourt T., Korczak D.J., Hewitt J.M.A., Pador P., Park J.L., McArthur B.A., Holy C., Neville R.D. (2023). Changes in Depression and Anxiety Among Children and Adolescents from Before to During the COVID-19 Pandemic: A Systematic Review and Meta-Analysis. JAMA Pediatr..

[17] Steinsbekk S., Ranum B., Wichstrøm L. (2022). Prevalence and Course of Anxiety Disorders and Symptoms from Preschool to Adolescence: A 6-Wave Community Study. J. Child Psychol. Psychiatry.

[18] McLaughlin K.A., Hatzenbuehler M.L. (2009). Stressful Life Events, Anxiety Sensitivity, and Internalizing Symptoms in Adolescents. J. Abnorm. Psychol..

[19] Monroe S.M., Harkness K.L. (2005). Life Stress, the “Kindling” Hypothesis, and the Recurrence of Depression: Considerations from a Life Stress Perspective. Psychol. Rev..

[20] Jenness J.L., Peverill M., King K.M., Hankin B.L., McLaughlin K.A. (2019). Dynamic Associations between Stressful Life Events and Adolescent Internalizing Psychopathology in a Multiwave Longitudinal Study. J. Abnorm. Psychol..

[21] Milas G., Ribar M., Ćavar F. (2025). Why Are Adolescent Girls More Prone to Stress-induced Depression? Testing Moderation, Mediation, and Reciprocal Causality in a Three-wave Longitudinal Study. J. Res. Adolesc..

[22] Monroe S.M., Simons A.D. (1991). Diathesis-stress Theories in the Context of Life Stress Research: Implications for the Depressive Disorders. Psychol. Bull..

[23] Hankin B.L. (2008). Cognitive Vulnerability-Stress Model of Depression during Adolescence: Investigating Depressive Symptom Specificity in a Multi-Wave Prospective Study. J. Abnorm. Child Psychol..

[24] Kendler K.S., Kessler R.C., Walters E.E., MacLean C., Neale M.C., Heath A.C., Eaves L.J. (2013). Stressful Life Events, Genetic Liability, and Onset of an Episode of Major Depression in Women. Depress. Sci. Ment. Health.

[25] Metalsky G.I., Abramson L.Y., Seligman M.E., Semmel A., Peterson C. (1982). Attributional Styles and Life Events in the Classroom: Vulnerability and Invulnerability to Depressive Mood Reactions. J. Pers. Soc. Psychol..

[26] Hastings P.D., Helm J., Mills R.S.L., Serbin L.A., Stack D.M., Schwartzman A.E. (2015). Dispositional and Environmental Predictors of the Development of Internalizing Problems in Childhood: Testing a Multilevel Model. J. Abnorm. Child Psychol..

[27] Belsky J., Bakermans-Kranenburg M.J., Van Ijzendoorn M.H. (2007). For Better and for Worse: Differential Susceptibility to Environmental Influences. Curr. Dir. Psychol. Sci..

[28] Post M. (1992). Transduction of Psychosocial Stress into the Neurobiology of Recurrent Disorder. Depression.

[29] Stroud C.B. (2020). The Stress Sensitization Model.

[30] Espejo E.P., Hammen C.L., Connolly N.P., Brennan P.A., Najman J.M., Bor W. (2007). Stress Sensitization and Adolescent Depressive Severity as a Function of Childhood Adversity: A Link to Anxiety Disorders. J. Abnorm. Child Psychol..

[31] Hammen C., Henry R., Daley S.E. (2000). Depression and Sensitization to Stressors among Young Women as a Function of Childhood Adversity. J. Consult. Clin. Psychol..

[32] Hammen C. (2005). Stress and Depression. Annu. Rev. Clin. Psychol..

[33] Hammen C. (2006). Stress Generation in Depression: Reflections on Origins, Research, and Future Directions. J. Clin. Psychol..

[34] Flynn M., Rudolph K.D. (2011). Stress Generation and Adolescent Depression: Contribution of Interpersonal Stress Responses. J. Abnorm. Child Psychol..

[35] March-Llanes J., Marqués-Feixa L., Mezquita L., Fañanás L., Moya-Higueras J. (2017). Stressful Life Events during Adolescence and Risk for Externalizing and Internalizing Psychopathology: A Meta-Analysis. Eur. Child Adolesc. Psychiatry.

[36] Kim K.J., Conger R.D., Elder G.H., Lorenz F.O. (2003). Reciprocal Influences between Stressful Life Events and Adolescent Internalizing and Externalizing Problems. Child Dev..

[37] Hamaker E.L., Kuiper R.M., Grasman R.P.P.P. (2015). A Critique of the Cross-Lagged Panel Model. Psychol. Methods.

[38] Mulder J.D. (2023). Power Analysis for the Random Intercept Cross-Lagged Panel Model Using the powRICLPM R-Package. Struct. Equ. Model. Multidiscip. J..

[39] OECD (2025). OECD Education and Skills in Croatia.

[40] Milas G., Klarić I.M., Malnar A., Šupe-Domić D., Slavich G.M. (2019). Socioeconomic Status, Social-Cultural Values, Life Stress, and Health Behaviors in a National Sample of Adolescents. Stress Health.

[41] Seiffge-Krenke I. (1995). Stress, Coping, and Relationships in Adolescence.

[42] Goodman R., Meltzer H., Bailey V. (1998). The Strengths and Difficulties Questionnaire: A Pilot Study on the Validity of the Self-Report Version. Int. Rev. Psychiatry Abingdon Engl..

[43] Vugteveen J., de Bildt A., Timmerman M.E. (2022). Normative Data for the Self-Reported and Parent-Reported Strengths and Difficulties Questionnaire (SDQ) for Ages 12–17. Child Adolesc. Psychiatry Ment. Health.

[44] Enders C.K. (2023). Missing Data: An Update on the State of the Art. Psychol. Methods.

[45] Cheung G.W., Rensvold R.B. (2002). Evaluating Goodness-of- Fit Indexes for Testing Measurement Invariance. Struct. Equ. Model. Multidiscip. J..

[46] Lucas R.E. (2023). Why the Cross-Lagged Panel Model Is Almost Never the Right Choice. Adv. Methods Pract. Psychol. Sci..

[47] Satorra A., Bentler P.M. (2001). A Scaled Difference Chi-Square Test Statistic for Moment Structure Analysis. Psychometrika.

[48] Hu L.T., Bentler P.M. (1999). Cutoff Criteria for Fit Indexes in Covariance Structure Analysis: Conventional Criteria versus New Alternatives. Struct. Equ. Model..

[49] Sivo S.A., Fan X., Witta E.L., Willse J.T. (2006). The Search for “Optimal” Cutoff Properties: Fit Index Criteria in Structural Equation Modeling. J. Exp. Educ..

[50] IBM (2020). IBM SPSS Statistics 27 Core System.

[51] Arbuckle J.L. (2020). IBM SPSS Amos 27 User’s Guide.

[52] Goodman A., Lamping D.L., Ploubidis G.B. (2010). When to Use Broader Internalising and Externalising Subscales Instead of the Hypothesised Five Subscales on the Strengths and Difficulties Questionnaire (SDQ): Data from British Parents, Teachers and Children. J. Abnorm. Child Psychol..

[53] Kenny D.A., Zautra A., Collins L.M., Sayer A.G. (2001). Trait–State Models for Longitudinal Data. New Methods for the Analysis of Change.

[54] Powers A., Casey B.J. (2015). The Adolescent Brain and the Emergence and Peak of Psychopathology. J. Infant Child Adolesc. Psychother..

[55] Lovibond P., Lovibond S. (1995). Pergamon the structure of negative emotional states: Scales (dass) with the beck depression and. Behav. Res. Ther..

[56] Griffith J.W., Zinbarg R.E., Craske M.G., Mineka S., Rose R.D., Waters A.M., Sutton J.M. (2010). Neuroticism as a Common Dimension in the Internalizing Disorders. Psychol. Med..

[57] Lahey B.B. (2009). Public Health Significance of Neuroticism. Am. Psychol..

[58] Borghuis J., Bleidorn W., Sijtsma K., Branje S., Meeus W.H.J., Denissen J.J.A. (2020). Longitudinal Associations between Trait Neuroticism and Negative Daily Experiences in Adolescence. J. Pers. Soc. Psychol..

[59] Lee J. (2011). Reciprocal Influences between Stress and Internalizing Problems in Korean Adolescents: A Cross-Lagged, Longitudinal Study. Asian J. Soc. Psychol..

[60] Goldstein B.L., Armeli S., Adams R.L., Florimon M.A., Hammen C., Tennen H. (2021). Patterns of Stress Generation Differ Depending on Internalizing Symptoms, Alcohol Use, and Personality Traits in Early Adulthood: A Five Year Longitudinal Study. Anxiety Stress Coping.

[61] Novak M., Parr N.J., Ferić M., Mihić J., Kranželić V. (2021). Positive Youth Development in Croatia: School and Family Factors Associated With Mental Health of Croatian Adolescents. Front. Psychol..

[62] Copeland W.E., Shanahan L., Costello E.J., Angold A. (2009). Childhood and Adolescent Psychiatric Disorders as Predictors of Young Adult Disorders. Arch. Gen. Psychiatry.

[63] Metz S.M., Frank J.L., Reibel D., Cantrell T., Sanders R., Broderick P.C. (2013). The Effectiveness of the Learning to BREATHE Program on Adolescent Emotion Regulation. Res. Hum. Dev..

[64] Ćavar Mišković F., Milas G. (2025). Mindfulness Reduces Adolescent Depression Through Stress Appraisal and Cognitive Reactivity: Evidence from a Four-Wave Longitudinal Study. Med. Lith..

[65] Garland E.L., Gaylord S.A., Fredrickson B.L. (2011). Positive Reappraisal Mediates the Stress-Reductive Effects of Mindfulness: An Upward Spiral Process. Mindfulness.

[66] Milas G., Ćavar F., Ribar M. (2024). How Much Stressful Life Events Really Matter? Conceptual and Methodological Difficulties in Assessing the Impact of Self-Reported Events on Adolescents’ Subjective Stress. Stress Health.

[67] Monroe S.M., Slavich G.M. (2020). Major Life Events. The Oxford Handbook of Stress and Mental Health.

