# Supplementary material for: Stress and Internalizing Problems in Adolescents: A Dynamic Longitudinal Analysis
Source: J Pers Med. 2025 Dec 8;15(12):612. doi: 10.3390/jpm15120612 (PMC12733919; doi:10.3390/jpm15120612)
Supplement: Supplementary file 1 [file jpm-15-00612-s001.zip › jpm-3954587-supplementary.pdf]

Table S1.

Binary logistic regression models predicting wave-specific missingness based on trait intensity assessed at a different wave

	B	S.E.	Wald	df	Sig.	Exp(B)	95% C.I.for EXP(B)	
							Lower	Upper
Subjective stress 1/2	.019	.080	.058	1	.810	1.020	.871	1.194
Subjective stress 1/3	.061	.080	.575	1	.448	1.062	.908	1.243
Subjective stress 2/1	-.076	.073	1.089	1	.297	.927	.803	1.069
Subjective stress 2/3	.025	.074	.117	1	.732	1.026	.888	1.185
Subjective stress 3/1	.102	.081	1.606	1	.205	1.108	.946	1.298
Subjective stress 3/2	-.039	.083	.223	1	.637	.961	.816	1.132
Emotional problems 1/2	.023	.025	.882	1	.348	1.024	.975	1.074
Emotional problems 1/3	.008	.026	.106	1	.745	1.008	.959	1.060
Emotional problems 2/1	-.027	.022	1.435	1	.231	.974	.932	1.017
Emotional problems 2/3	-.002	.023	.011	1	.917	.998	.953	1.044
Emotional problems 3/1	.012	.025	.245	1	.621	1.012	.965	1.062
Emotional problems 3/2	-.011	.025	.186	1	.667	.989	.941	1.040
Peer problems 1/2	-.029	.023	1.437	1	.221	.974	.912	1.027
Peer problems 1/3	-.080	.037	4.661	1	.031	.923	.858	.993
Peer problems 2/1	-.076	.074	1.087	1	.295	.927	.806	1.079
Peer problems 2/3	-.068	.033	4.127	1	.042	.934	.875	.998
Peer problems 3/1	.071	.037	3.738	1	.053	1.073	.999	1.153
Peer problems 3/2	.059	.036	2.629	1	.105	1.061	.988	1.139

Note. Significance was evaluated using a Bonferroni-corrected threshold of p = .0028 (.05/18).

Table S2.

Evaluation of the missing-at-random (MAR) assumption using regressions of Wave 2 and Wave 3 missingness indicators on sex, socioeconomic status (SES), and school track.

Subjective stress t2

Predictor	B	SE B	β	p	R^2
Sex (male = 1, female=2)	-.034	.025	-.036	.173	
School track (vocational=1, gymnasium=2)	.313	.026	.322	.000	0.114***
SES	.037	.014	.068	.010	

Subjective stress t3

Predictor	B	SE B	β	p	R^2
Sex (male = 1, female=2)	-.032	.024	-.038	.179	
School track (vocational=1, gymnasium=2)	-.068	.024	-.079	.005	0.011**
SES	-.018	.013	-.037	.183	

Emotional problems t2

Predictor	B	SE B	β	p	R^2
Sex (male = 1, female=2)	-.034	.025	-.036	.173	
School track (vocational=1, gymnasium=2)	.316	.026	.325	.000	0.115***
SES	.036	.014	.068	.011	

Emotional problems t3

Predictor	B	SE B	β	p	R^2
Sex (male = 1, female=2)	-.033	.024	-.039	.160	
School track (vocational=1, gymnasium=2)	-.077	.025	-.089	.002	0.012**
SES	-.016	.013	-.033	.238	

Peer problems t2

Predictor	B	SE B	β	p	R^2
Sex (male = 1, female=2)	-.034	.025	-.036	.173	
School track (vocational=1, gymnasium=2)	.316	.026	.325	.000	0.115***
SES	.036	.014	.068	.011	

Peer problems t3

Predictor	B	SE B	β	p	R^2
Sex (male = 1, female=2)	-.033	.024	-.039	.160	
School track (vocational=1, gymnasium=2)	-.077	.025	-.089	.002	0.012**
SES	-.016	.013	-.033	.238	

*Note: ** $p < .001$; *** $p < .001$*

Table S3.

Measurement Invariance Across Sex for Subjective Stress, Emotional Problems, and Peer Problems

	Model	χ^2	df	p	CFI	RMSEA	$\Delta\chi^2$	df	p	ΔCFI	ΔRMSEA	Invariance
Subjective stress (PQ)	Configural	165.753	18	< .001	0.953	0.112						
	Metric	181.15	23	< .001	0.95	0.103	15.397	5	.009	0.003	-0.009	Yes
	Scalar	184.541	28	< .001	0.95	0.093	3.391	5	.640	0.000	-0.010	Yes
Emotional problems (SDQ)	Configural	31.441	10	< .001	0.992	0.058						
	Metric	31.429	14	< .001	0.994	0.044	-0.012	4	-	-0.002	-0.014	Yes
	Scalar	37.734	18	< .001	0.993	0.041	6.305	4	.177	0.001	-0.003	Yes
Peer problems (SDQ)	Configural	41.341	10	< .001	0.941	0.070						
	Metric	42.835	14	< .001	0.945	0.056	1.494	4	.828	-0.004	-0.014	Yes
	Scalar	50.096	18	< .001	0.939	0.052	7.261	4	.123	0.006	-0.004	Yes

Note. df = degrees of freedom; CFI = Comparative Fit Index; RMSEA = Root Mean Square Error of Approximation.

Table S4.

Longitudinal Measurement Invariance for Subjective Stress, Emotional Problems, and Peer Problems

	Model	χ^2	df	p	CFI	RMSEA	$\Delta\chi^2$	df	p	ΔCFI	ΔRMSEA	Invariance
Subjective stress (PQ)	Configural	396.024	27	< .001	0.96	0.104						
	Metric	407.763	37	< .001	0.96	0.089	11.739	10	.303	0.000	-0.015	Yes
	Scalar	468.735	47	< .001	0.954	0.084	60.972	10	.000	0.006	-0.005	Yes
Emotional problems (SDQ)	Configural	46.001	15	< .001	0.996	0.040						
	Metric	43.665	23	< .001	0.997	0.027	-2.336	8	-	-0.001	-0.013	Yes
	Scalar	62.468	31	< .001	0.996	0.028	18.803	8	.016	0.001	0.001	Yes
Peer problems (SDQ)	Configural	90.066	15	< .001	0.955	0.063						
	Metric	89.39	23	< .001	0.960	0.048	-0.676	8	-	-0.005	-0.015	Yes
	Scalar	118.047	31	< .001	0.948	0.047	28.657	8	.000	0.012	-0.001	Marginal

Note. df = degrees of freedom; CFI = Comparative Fit Index; RMSEA = Root Mean Square Error of Approximation.

Table S5.

Model Fit Comparison Between Unconstrained and Constrained (Autoregressive and Cross-Lagged Paths) RI-CLPM Models: Subjective Stress and Emotional Problems

Model	χ^2	df	p	CFI	TLI	RMSEA	$\Delta\chi^2$	df	p	ΔCFI	ΔTLI	ΔRMSEA
Unconstrained	1.662	1	0.197	1.000	0.997	0.020						
Constrained	9.425	5	0.093	0.999	0.996	0.023	7.763	4	0.002	0.001	0.001	0.003

Note. df = degrees of freedom; CFI = comparative fit index; TLI= Tucker–Lewis Index; RMSEA = root-mean-square error of approximation.

Table S6.

Model Fit Comparison Between Unconstrained and Constrained (Autoregressive and Cross-Lagged Paths) RI-CLPM Models: Subjective Stress and Peer Problems

Model	χ^2	df	p	CFI	TLI	RMSEA	$\Delta\chi^2$	df	p	ΔCFI	ΔTLI	ΔRMSEA
Unconstrained	3.132	1	0.077	0.999	0.984	0.036						
Constrained	13.47	5	0.019	0.997	0.987	0.032	10.338	4	0.002	0.002	-0.003	-0.004

Note. df = degrees of freedom; CFI = comparative fit index; TLI= Tucker–Lewis Index; RMSEA = root-mean-square error of approximation.

Table S7.

Random Intercept Cross-Lagged Panel Model (RI-CLPM) Specification for Stress and Emotional Problems

Component	Specification
Model type	RI-CLPM with three waves
Random intercepts	Latent intercept factors for Stress and Emotional Problems (EP)
Autoregressive paths	EP2 ← EP1 = EP3 ← EP2 (constrained equal); STR2 ← STR1 = STR3 ← STR2 (constrained equal)
Cross-lagged paths	Stress → EP: EP2 ← STR1 = EP3 ← STR2 (constrained equal); EP → Stress: STR2 ← EP1 = STR3 ← EP2 (constrained equal)
Within-wave covariances	Residual correlations estimated where applicable
Estimator	ML with FIML

Table S8.

Parameter Estimates for the RI-CLPM: Stress and Emotional Problems

Parameter	B	SE	95% CI	β	p	R^2
**Autoregressive paths**						
EP2 ← EP1	.169	.065	[.042, .296]	.167	.009	.045
EP3 ← EP2	.169	.065	[.042, .296]	.179	.009	.048
STR2 ← STR1	.235	.075	[.088, .382]	.244	.002	.080
STR3 ← STR2	.235	.075	[.088, .382]	.227	.002	.067
**Cross-lagged paths**						
EP2 ← STR1	.244	.197	[–.142, .630]	.073	.215	—
EP3 ← STR2	.244	.197	[–.142, .630]	.075	.215	—
STR2 ← EP1	.020	.016	[–.011, .051]	.069	.208	—
STR3 ← EP2	.020	.016	[–.011, .051]	.067	.208	—
**Intercepts**						—
Stress Wave 1	2.211	.021	[2.170, 2.252]	—	< .001	—
Stress Wave 2	2.340	.021	[2.299, 2.381]	—	< .001	—
Stress Wave 3	2.123	.021	[2.083, 2.164]	—	< .001	—
EP Wave 1	3.791	.069	[3.656, 3.926]	—	< .001	—
EP Wave 2	3.989	.070	[3.852, 4.126]	—	< .001	—
EP Wave 3	3.808	.067	[3.677, 3.939]	—	< .001	—
**Covariances**						—
EP1 ↔ STR1	.381	.054	[.275, .487]	.467	< .001	—
STR_trait ↔ EP_trait	1.044	.073	[.900, 1.188]	.802	< .001	—
e1 ↔ e2	.252	.058	[.139, .365]	.340	< .001	—
e3 ↔ e4	.257	.035	[.188, .326]	.354	< .001	—
**Variances**						—
EP1 Residual	2.687	.202	[2.291, 3.083]	—	< .001	—
STR1 Residual	.247	.021	[.206, .288]	—	< .001	—
EP Trait	4.174	.263	[3.658, 4.690]	—	< .001	—
STR Trait	.406	.027	[.353, .459]	—	< .001	—
EP Residuals e1/e2/e3	2.611 / .211 / .229	—	—	—	—	—
STR Residual e4	2.297	.148	[2.007, 2.587]	—	< .001	—

Table S9.

Random Intercept Cross-Lagged Panel Model (RI-CLPM) Specification for Stress and Peer Problems

Component	Specification
Model type	RI-CLPM with three waves
Random intercepts	Latent intercept factors for Stress and Peer Problems (PP); factor loadings fixed to 1
Autoregressive paths	PP2 ← PP1 = PP3 ← PP2 (constrained equal); STR2 ← STR1 = STR3 ← STR2 (constrained equal)
Cross-lagged paths	Stress → PP: PP2 ← STR1 = PP3 ← STR2 (constrained equal); PP → Stress: STR2 ← PP1 = STR3 ← PP2 (constrained equal)
Within-wave residual covariances	Estimated where supported
Means	Means of random intercepts freely estimated
Variances	Latent residual variances freely estimated
Estimator	Maximum likelihood with FIML for missing data

Table S10.

Parameter Estimates for the RI-CLPM: Stress and Peer Problems

Parameter	B	SE	95% CI	β	p	R^2
**Autoregressive paths**						
PP2 ← PP1	.249	.060	[.132, .366]	.237	< .001	.059
PP3 ← PP2	.249	.060	[.132, .366]	.259	< .001	.072
STR2 ← STR1	.269	.076	[.120, .418]	.275	< .001	.076
STR3 ← STR2	.269	.076	[.120, .418]	.264	< .001	.071
**Cross-lagged paths**						
PP2 ← STR1	.079	.146	[–.207, .365]	.029	.589	—
PP3 ← STR2	.079	.146	[–.207, .365]	.029	.589	—
STR2 ← PP1	.002	.019	[–.035, .039]	.005	.929	—
STR3 ← PP2	.002	.019	[–.035, .039]	.005	.929	—
**Intercepts**						—
Stress Wave 1	2.211	.021	[2.170, 2.252]	—	< .001	—
Stress Wave 2	2.340	.021	[2.299, 2.381]	—	< .001	—
Stress Wave 3	2.124	.021	[2.083, 2.165]	—	< .001	—
PP Wave 1	2.221	.047	[2.129, 2.313]	—	< .001	—
PP Wave 2	2.386	.050	[2.288, 2.484]	—	< .001	—
PP Wave 3	2.512	.047	[2.420, 2.604]	—	< .001	—
**Covariances**						—
PP1 ↔ STR1	.092	.037	[.019, .165]	.145	.012	—
STR_trait ↔ PP_trait	.339	.044	[.253, .425]	.439	< .001	—
e1 ↔ e2	.165	.038	[.090, .240]	.268	< .001	—
e3 ↔ e4	.102	.026	[.051, .153]	.171	< .001	—
**Variances**						—
PP1 Residual	1.657	.126	[1.410, 1.904]	—	< .001	—
STR1 Residual	.246	.021	[.205, .287]	—	< .001	—
PP Trait	1.469	.132	[1.209, 1.729]	—	< .001	—
STR Trait	.404	.027	[.351, .457]	—	< .001	—
PP Residuals e1/e2/e3	1.732 / .218 / .227	—	—	—	—	—
STR Residual e4	1.584	.091	[1.405, 1.763]	—	< .001	—
